# Recent Advances in Self-Assembled Molecular Application in Solar Cells

**DOI:** 10.3390/nano14090779

**Published:** 2024-04-30

**Authors:** Linkun Zhong, Chuangping Liu, Shi Lai, Bing’e Li, Baihong Zheng, Xiaoli Zhang

**Affiliations:** Guangdong Provincial Key Laboratory of Information Photonics Technology, School of Physics and Opto-Electronic Engineering, Guangdong University of Technology, Guangzhou 510006, China; 2112315022@mail2.gdut.edu.cn (L.Z.); 2112315025@mail2.gdut.edu.cn (C.L.); 18565578056@163.com (S.L.); leebange@163.com (B.L.); bhpercival@163.com (B.Z.)

**Keywords:** perovskite solar cells, self-assembled monolayers, power conversion efficiency, energy level, application

## Abstract

Perovskite solar cells (PSCs) have attracted much attention due to their low cost, high efficiency, and solution processability. With the development of various materials in perovskite solar cells, self-assembled monolayers (SAMs) have rapidly become an important factor in improving power conversion efficiency (PCE) due to their unique physical and chemical properties and better energy level matching. In this topical review, we introduced important categories of self-assembled molecules, energy level modulation strategies, and various characteristics of self-assembled molecules. In addition, we focused on reviewing the application of self-assembled molecules in solar cells, and explained the changes that self-assembled molecules bring to PSCs by introducing the mechanism and effect of self-assembled molecules. Finally, we also elaborated on the challenges currently faced by self-assembled molecules and provided prospects for their applications in other optoelectronic devices.

## 1. Introduction

**Figure 1:**
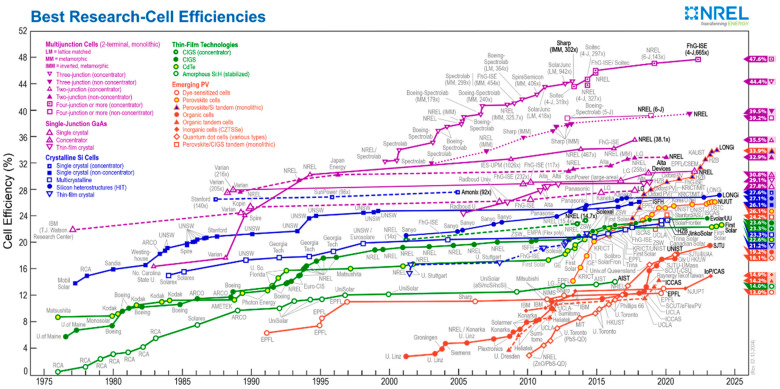
Development of solar cell efficiency [18].

After fossil fuels, solar energy has become another important energy source due to its renewable and environmentally friendly characteristics. Since the 1970s, the transmission efficiency of solar cells has achieved a qualitative leap (Figure 1). In the past decade, perovskite solar cells (PSCs) have drawn tremendous attention because of their high power conversion efficiency, low cost, and solution processing characteristics [1,2,3,4,5,6]. Recently, a certified PCE of 26.14% has been reported, similar to the PCE of silicon-based solar cells [7].

**Figure 2:**
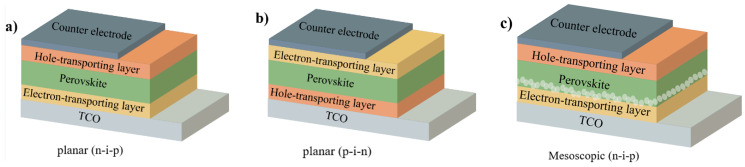
Schematic diagrams of common PSC device structures (**a**) planar (n-i-p) PSC, (**b**) planar (n-i-p) PSC and (**c**) mesoscopic (n-i-p) PSC. TCO = transparent conductive oxide.

The structural formula of perovskite is ABX_3_, where A stands for a monovalent cation (such as formamidinium, FA^+^; methylammonium, MA^+^, etc.), B stands for a metal ion (such as plumbum ion, Pb^2+^; tin ion, Sn^2+^; zinc ion, Zn^2+^, etc.), and X is generally a halide anion (such as chloride, Cl^−^; bromide, Br^−^; iodide, I^−^, etc.) [8,9,10]. Typical PSCs contain hole-transport layers (HTLs), perovskite-based absorber layers, and electron-transporting layers (ETLs) [11]. There are three types of perovskite solar cell devices: mesoporous n-i-p, planar n-i-p, and planar p-i-n structure (Figure 2). After the perovskite absorption layer absorbs photons, the electron-hole pairs generated by the light are dissociated into electrons and holes by the built-in electric field. The electrons are transferred to the negative electrode through ETL, and the holes are transferred to the positive electrode through HTL [12]. In formal devices, the commonly used choice for the ETL is usually metal oxides such as SnO_2_ or TiO_2_, while the HTL typically uses polymers such as poly2,4,6-trimethyl-N, N-diphenylamine (PTAA), or organic semiconductor materials such as 2,2′,7,7′-tetra (N,N-dip-methoxyaniline)-9,9′- spirodifluorene (spiro-OMeTAD) [13,14]. In trans devices, the selection of ETL is usually based on fullerene derivatives such as [6]—phenyl-C71-butyric acid methyl ester (PCBM), while HTL is typically poly(3,4-ethylenedioxythiophene):polydimethylene sulfonate (PEDOT:PSS) [15,16,17]. However, in reverse devices, using PEDOT:PSS as the device PCE for HTL is unsatisfactory. Therefore, a new type of self-assembled monolayers is used in HTL to achieve efficient hole extraction and transmission, greatly improving the efficiency of the device.

Self-assembled monolayers have received widespread attention in recent years because of their excellent performance. For SAM, their molecular structure is relatively simple and the film thickness is much smaller, so the use of self-assembled molecules can reduce the cost of PSCs [19,20,21,22,23,24]. Due to its small thickness, SAM can reduce parasitic absorption and overcome resistive loss, thereby improving the performance of PSCs [21,25]. Compared with ordinary PSCs, HOMO energy levels can be adjusted by selecting different self-assembled molecules according to the desired properties, thereby achieving band matching with various bandgap perovskites [20]. In addition, SAM can also avoid solution-based thin film deposition during film formation, resulting in a more uniform film on a large area [26]. Based on these excellent performances, SAM plays an undeniable role in improving device efficiency and also lays the foundation for the application of self-assembled molecules in other related fields.

In this topical review, we discuss the basic characteristics of SAM, SAM application in solar cells, and the challenges and opportunities of SAM. We first introduce the structure of SAM, the synthesis of SAM, and the characteristics of SAM. Subsequently, we elaborated on the applications of SAM as HTL and ETL in different solar cells, such as perovskite SCs, organic SCs, and dye-sensitized SCs. Finally, we elaborated on the challenges and opportunities that SAM currently faces in terms of its application.

## 2. Self-Assembled Monolayers

### 2.1. Structure

Usually, SAMs compose of aromatic amines, benzene rings, pyridine, thiophene, fullerene, and various anchoring groups. In addition, by replacing certain atoms on SAMs with halogens, the performance of solar cells can be enhanced to a certain extent, the ability to move holes/carriers can be increased, the crystal resistance can be reduced, trap recombination can be suppressed, and the carrier lifetime can be improved [27]. SAMs can be classified based on the characteristics of their different parts. In Table 1, we list several typical SAMs, showcasing their structures, molecular types, passivation layer types, and their respective advantages in application.

### 2.2. Energy Level

**Figure 3:**
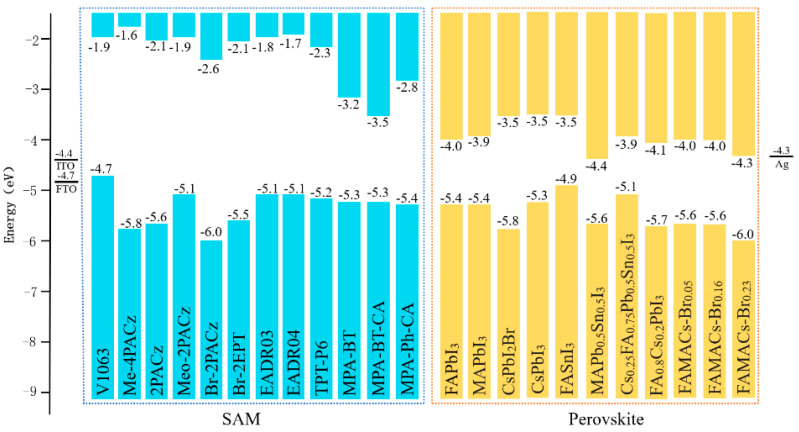
Energy level of SAMs, including V1036 [19]; Me-4PACz [51]; 2PACz [51]; MeO-2PACz [52]; Br-2PACz [27]; Br-2EPT [53]; EADR03, EADR04 [53]; TPT-P6 [24]; MPA-BT, MPA-BT-CA [30]; MPA-Ph-CA [25]. Energy level arrangement of common perovskite components: FAPbI_3_, MAPbI_3_, CsPbI_2_Br, CsPbI_3_, FASnI_3_, MAPb_0.5_Sn_0.5_I_3_, Cs_0.25_FA_0.75_Pb_0.5_Sn_0.5_I_3_, FA_0.8_Cs_0.2_PbI_3_, FAMACs-Br_0.05_, FAMACs-Br_0.16_, FAMACs-Br_0.23_ [26].

To achieve efficient charge collection and excellent optoelectronic performance at the interface, matching energy levels is crucial [46,47,48]. Self-assembling molecules have more convenient energy level modulation compared to traditional inorganic compounds and polymers (such as NiO, PTAA, PEDOT:PSS, and PDCBT), and the required performance can be achieved through the design of different parts of the molecule (Figure 3). The regulation of the donor part has a better ability to regulate the HOMO energy level of the molecule than the change of the anchoring group. By combining different aromatic amines, it can achieve band alignment matching with different bandgap perovskites. Not only the molecular energy levels, but also the interface dipoles formed through the molecular layer are tools for regulating energy level matching at the interface, and can also serve as a tool to control the work function of TCO [29,49,50].

### 2.3. Characteristics of SAMs

In the field of interface modification, self-assembled thin films have become one of the mainstream materials [54]. The principle of self-assembled molecules is to attach molecules to a substrate through chemical adsorption, thereby spontaneously assembling a single-layer thin film with a long range order. Similar to traditional two-dimensional materials such as graphene, the performance of self-assembled films is closely related to their film thickness. Apart from the key factor of film thickness, the thickness and surface roughness of the substrate determine the difficulty of SAM in forming films with regular and defect-free growth. From the perspective of intermolecular bonding strength, traditional two-dimensional materials are connected by covalent bonds, and their bonding strength is much higher than the π-π interactions and Van der Waals forces between SAM molecules. Therefore, the density and regularity of self-assembly differ significantly from two-dimensional materials. Although the unit density of SAM is relatively low, the advantage it brings is that it can be attached by solution or vacuum evaporation methods and can be directly modified by precursor molecules.

Due to the fact that SAM molecules attach to the substrate through chemical adsorption, agglomeration is prone to occur during the adsorption process. To avoid excessive aggregation of SAM molecules, it is necessary to remove excess molecules through certain solvent cleaning. In addition, SAM’s selection and extraction of charge carriers are highly dependent on energy level alignment, which is related to the levels of short circuit current density (J_oc_), open circuit voltage (V_oc_), and field factor (FF). The structure of SAM molecules themselves is also quite important, and the effects of interface defect passivation and active layer activity regulation caused by different functional groups replacing different head molecules vary greatly, thereby affecting surface coverage, uniformity, and crystallinity.

The impact of self-assembly on the stability of devices is mainly manifested in their own chemical stability, mechanical stability, and thermal stability. Stable components such as alkane chains in self-assembled molecules can serve as barriers at the interface, preventing chemical reactions and improving device stability. Generally speaking, the mechanical stress in thin film electronic devices formed by solution processing can seriously reduce the stability of the device. The Van der Waals bonds and π-π interactions formed by self-assembled molecules belong to soft connections, so self-assembly has a high tolerance to mechanical deformation and even self-healing.

## 3. SAM Application in Solar Cells

### 3.1. Application in Perovskite Solar Cells

Researchers have found that self-assembled molecules have a good passivation effect on electron traps on ITO and FTO, and molecular chain length and head structure are important factors affecting charge motion and passivation kinetics [55]. Through chemical bonding contact with ETL or HTL, the work function of HTL or ETL can be adjusted by SAMs to better match the energy level of perovskite [56]. SAMs can more effectively extract electrons from perovskite when in contact with it, efficiently improving current density [57].

**Figure 4:**
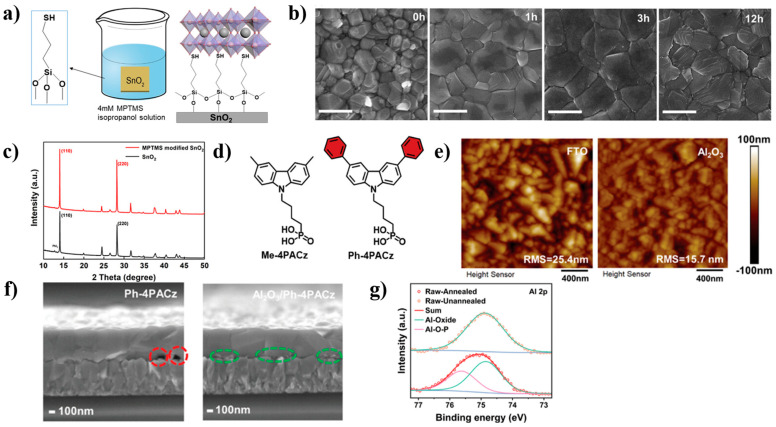
(**a**) Molecular structure, modification methods, and binding diagrams of MPTMS; (**b**) SEM images of perovskite thin films before and after treatment; (**c**) XRD comparison of perovskite thin films [59]; (**d**) Schematic diagram of Ph-4PACz molecular structure; (**e**) Comparison of the AFM of the substrate before and after adding Al_2_O_3_; (**f**) SEM images of perovskite films formed on Me-4PACz and Ph-4PACz; (**g**) Al 2p spectra of FTO/Al_2_O_3_-NPs/Ph-4PACz annealed or unannealed [28].

Dai et al. reported that iodine-terminated self-assembled monolayers (I-SAMs) can increase the adhesion toughness between the electronic transport layer (ETL) and the halide perovskite film interface by 50%, thereby improving mechanical reliability and reducing hysteresis, ultimately improving the operational stability of the device [58]. As shown in Figure 4a, Shi et al. developed a 3-mercaptopropyltrimethoxysilane (MPTMS) SAM which can improve the crystallization quality and surface morphology of sequential perovskite thin films. In the process of two-step device preparation, this molecule can not only slow down the growth rate of perovskite crystals, but can also passivate the SnO_2_/perovskite interface, suppress carrier recombination, and improve the extraction rate of photo-generated electrons [59]. However, with the continuous extension of processing time, the thin film begins to become rough and is accompanied by a decrease in grain size. This is because the presence of excessive MPTMS can hinder crystal growth. From the XRD characterization (Figure 4c), it can be seen that after the introduction of MPTMS, the peak intensities at (110) and (220) are significantly increased, further indicating that the influence of MPTMS on the membrane is beneficial.

In previous studies, the optimization effect of Me-4PACz in p-i-n devices has been confirmed, and based on this, a novel SAM molecule, Ph-4PACz (Figure 4d), was further studied. Research has shown that the band alignment of perovskite can be altered by the high polarity of Ph-4PACz, and the energy loss will also be reduced accordingly. However, in large-area devices, the filling factor (FF) will be significantly reduced. However, when aluminum nanoparticles (Al_2_O_3_ NPs) are introduced into the interface between Ph-4PACz and rough FTO, from the AFM results (Figure 4e), it can be seen that after adding Al_2_O_3_-NPs, the surface roughness of the film has significantly decreased. Al_2_O_3_-NPs have filled the existing gaps, and the formation of (Al-O-P) further proves the combination of Ph-4PACz and Al_2_O_3_-NPs. Under this process, a small area PCE of 25.60% can be achieved, and it can also reach 24.61% (certified at 24.48%) on a large area (1 cm^2^) [28].

Regulating the deposition of bipolar self-assembled molecules on the surface of perovskite films is an effective method to improve work function (WF). Canil et al. utilized the direction of surface dipoles to regulate the displacement direction of valence and energy bands in perovskite, and the displacement magnitude was tuned by adjusting the density of dipoles [46]. In wide bandgap (WBG) PSCs, the ultra-thin SAM between HTLs and perovskite thin films provides the optimal network connection, which improves the carrier lifetime and ion migration activation energy. The PCE of these devices can reach 20.4%, and their resistance to wet and thermal stress stability is also enhanced with increasing hydrophobicity SAM in active layers [60]. Similarly, in tin perovskite solar cells, perovskite thin films can also be deposited on the surface of SAM with added dye sensitizers. In a recent study, researchers compared cyano/cyano or cyano/carboxylic acid groups coupled with a phenyl ring or thiophene unit, which can effectively provide electrons to perovskite and maintain good stability of the device [61].

**Figure 5:**
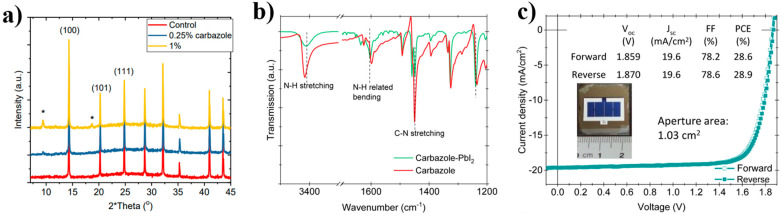
(**a**) Comparison of XRD before and after adding carbazole, and * in the curve indicates carbazole signal; (**b**) FTIR spectroscopy of pure carbazole and carbazole-PbI_2_ films; (**c**) J-V curves of the champion tandem cell with an aperture area of 1.03 cm^2^ [62].

In addition to acting independently in the device, SAM can also be doped into other layers to optimize the device. Liu et al. reported that carbazole (PTAA, 2PACz) was added to the perovskite precursor [62], after which the peak intensities at (100) and (101) were enhanced, indicating that the addition of carbazole can promote the preferred growth of crystals. It can also be seen from the FAIR of pure carbazole and carbazole PbI_2_ films (Figure 5b) that the interaction between carbazole and perovskite through N-H bonds is mainly reflected in the bending vibration displacement and N-H stretching at 3410 cm^−1^ and 1602 cm^−1^. This type of device which integrates a wide band-gap perovskite cell onto a SHJ bottom cell achieved a certification efficiency of 28.6% (Figure 5c), and significant results were also achieved in subsequent stability tests.

The role of the electronic transfer layer (ETL) in devices is crucial for solar cells. The ETL should be relatively thin to reduce resistance loss, and the collector area should also be continuously and uniformly covered during the film formation process. As the device area increases, the difficulties brought about by achieving these conditions will gradually increase. Therefore, using SAMs as hole transport layers is a very good choice.

**Figure 6:**
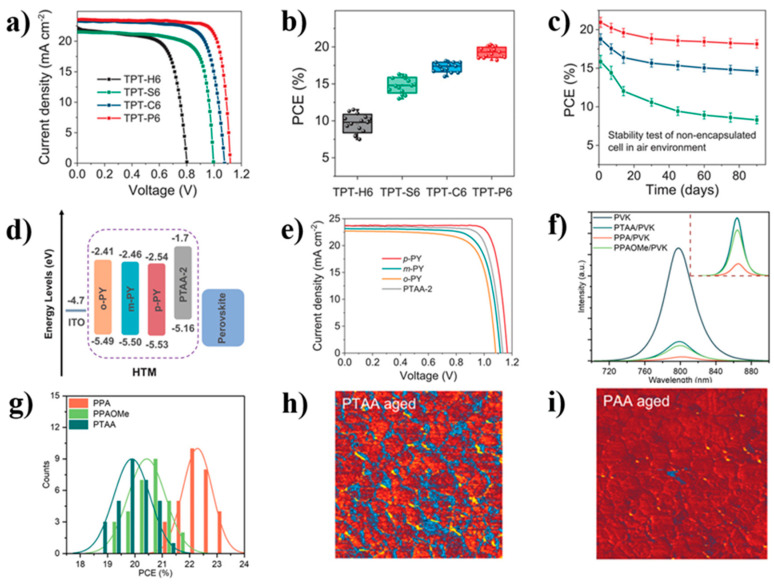
(**a**) J-V curves under different anchoring groups; (**b**) Distribution of PCE under different anchoring groups; (**c**) Stability of different PSCs prepared in the air [24]; (**d**) Energy level arrangement of PTAA and substituted molecules; (**e**) J-V curves of devices using four different molecules [63]; (**f**) Steady-state PL spectrum of the perovskite films deposited on glass; (**g**) Efficiency distribution of 20 devices based on different molecules; (**h**) PTAA and (**i**) PAA AFM-IR images of devices aged under illumination and 55 °C, 500 h in N_2_ atmosphere conditions [31].

In SAM materials, different anchoring groups have different bonding strengths, which in turn affect the strength of the monolayer. In previous studies, researchers designed three different self-assembled molecules with anchoring groups (-SO_3_H, -COOH, and -PO_3_H_2_) compared with molecules without anchoring groups. It was demonstrated that anchoring groups with high binding strength can increase the assembly rate and density of molecules, thereby improving the conversion efficiency of PSCs [24]. From the perspectives of photoelectric characteristics, PCE distribution, and stability (Figure 6a–c), TPT-P6 (-PO_3_H_2_) outperforms several other anchoring groups in all aspects. The characterizations have successfully demonstrated that the phosphate-anchored group (-PO_3_H_2_) has higher binding strength compared to other groups.

Similarly, due to the low HOMO levels, hydrophobicity, and absence of passivation groups in the traditional hole transport layer material PTAA, a series of novel molecules were developed based on traditional commercial PTAA molecules [63]. Alkyl groups were removed from the traditional commercial PTAA end chains and replaced with different pyridine units, resulting in deep HOMO levels and passivation groups. From the energy level arrangement (Figure 6d), it can be seen that the energy level arrangement of the substituted molecules matches more closely with the energy levels of perovskite. The experimental results (Figure 6e) show that p-PY is the best-performing molecule among the three substituted PTAA and PSCs; this molecule was used as a hole transport material and achieved a 22% efficiency (0.09 cm^2^).

In previous studies, (-PO_3_H_2_) was confirmed to have better binding strength than other functional groups, so designing molecules based on (-PO_3_H_2_) is one of the current research focuses. Guo et al. synthesized two novel self-assembled hole transport materials. The advantage of triphenylamine in these two molecules is that the energy level alignment between ITO and perovskite becomes more convenient, leading to improved hole extraction and electron-blocking effects [31]. As shown in Figure 6f, the PL quenching at the PPA/perovskite interface is very significant, which means that the efficiency of the dissociated holes in the perovskite is greatly improved during PPA extraction. In the PCE distribution diagram of the device (Figure 6g), it can also be seen that the PCE of PPA-based devices is generally higher than that of PPAOMe and PTAA. After treatment with ISOS-L^−1^ condition for 1000 h (Figure 6h,i), PTAA showed significant changes, with only a small portion of PPA undergoing changes. On this basis, the device obtained 23.24% PCE.

**Figure 7:**
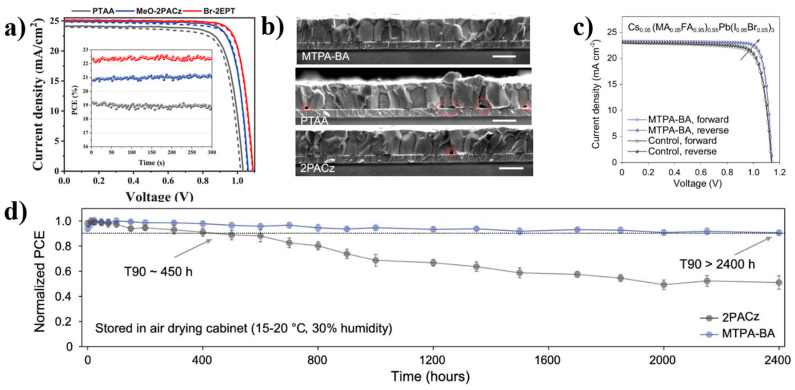
(**a**) Comparison of J-V curves of PTAA, MeO-2PACz, and Br-2EPT [64]; (**b**) Cross-section SEM images of ITO/MTPA-BA, PTAA, and 2PACz/perovskite; (**c**) J-V curves based on different HSCs; (**d**) PSC stability testing based on 2PACz and MTPA-BA [25].

In addition to excellent performance, cost is also an important constraint for material applications. Ullah et al. have launched a highly cost-effective SAM Br-2EPT. Not only does this new type of molecule not require precious metals as catalysts, but it also uses environmentally friendly low-cost materials as precursors, so the synthesis of this material is only 31.48 $ g^−1^. In comparison with traditional materials PTAA and self-assembled molecule MeO-2PACz, as shown in Figure 7a, Br-2EPT also exhibits impressive performance, with the champion device obtaining 22.44% PCE (certified 21.81%) [64]. Furthermore, the anchoring groups in SAM molecules can cause certain corrosion to the substrate, thereby affecting the stability of the device. Guo et al. proposed a series of boronic acid-substituted aromatic amines as boronic acid anchoring groups SAM (TPA-BA, MTPA-BA, MeOTPA-BA). From the cross-sectional SEM images of ITO/MTPA-BA, PTAA, and 2PACz/perovskite (Figure 7b), it can be seen that there are some gaps between PTAA and 2PACz and the substrate, while MPTA-BA is closely connected to the substrate [25]. Due to the improvement of perovskite substrate contact and buried passivation by the borate group SAM, from the J-V curve and stability curve (Figure 7c,d), it can be seen that this type of device achieved 23% PCE, with stability of about 5 times that of the phosphate group SAM (T90 of 2PACZ is about 450 h, and T90 of MTPA-BA is about 2400 h).

**Figure 8:**
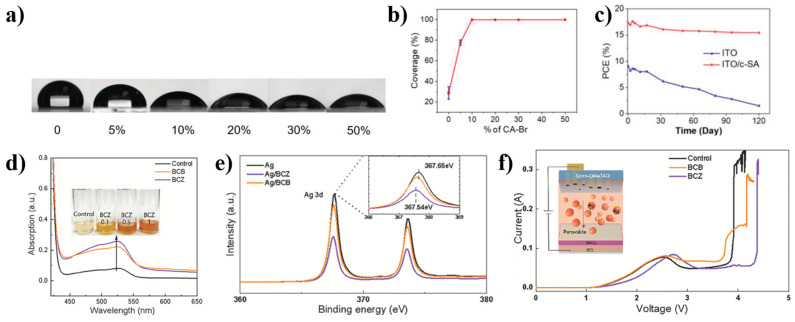
(**a**) The effect of different content of CA-Br on the contact angle of solutions; (**b**) Surface affinity curves of perovskite thin films and substrates; (**c**) 30% humidity stability test under one sunlight intensity [26]; (**d**) UV-vis absorption of the impact of BCZ content on HTL; (**e**) XPS of Ag 3d in the Ag film deposited on the Spiro-OMeTAD, BCB-Spiro-OMeTAD, and BCZ-Spiro-OMeTAD films; (**f**) I-V curves of devices based on different HTL materials [66].

One of the most effective methods to improve the uniformity and wettability of the hole transport layer is the anchoring-based co-assembly (ACA) strategy. Researchers obtained a highly wettable uniform hole transport layer by synergistic coupling of a hydrodynamic ammonium salt CA-Br with hole-transporting triphenylamine derivatives. From the influence of CA-Br on the wettability of self-assembly (Figure 8a), the wetting angle of TPA-PT-C6 decreases from the initial 101.8° to 64.6° with the addition of CA-Br, which greatly reduces the difficulty of perovskite deposition. From the optoelectronic characteristics of devices with different concentrations of CA Br, it can also be seen that PCE reaches its peak at 30% CA Br, and further addition of concentration will reduce PCE. From the surface affinity curves of the perovskite film and substrate, it can be seen that when the amount of CA-Br added is only 5%, the film coverage increases from 20% to 70%. When the amount of CA-Br reaches 10%, 100% coverage of the perovskite film can be achieved (Figure 8b), and the stability under this method has also been significantly improved (Figure 8c) [26]. A conversion efficiency of 12.67% was achieved in modules with an aperture area of 36 cm^2^. 

**Figure 9:**
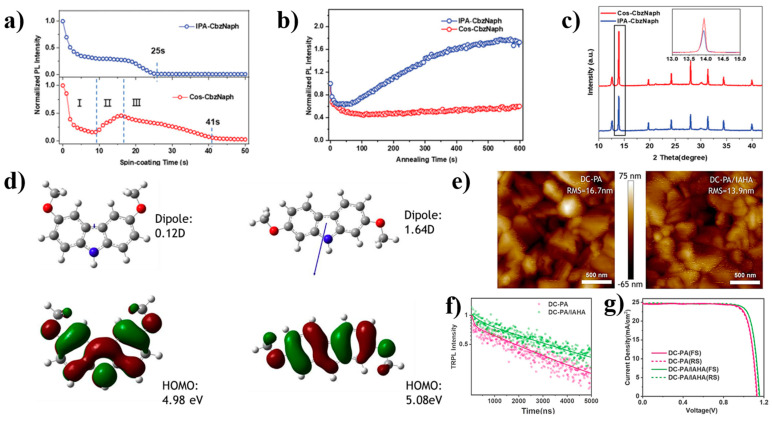
(**a**) PL spectra during spin coating process; (**b**) PL spectra during annealing process; (**c**) XRD patterns of perovskite thin films deposited on different HTLs [52]; (**d**) The molecular structures, dipoles, and HOMO levels of MeO-2PACz and DCPA; (**e**) AFM images of the surface of perovskite thin films; (**f**) I-V curves of devices based on different HTL materials; (**g**) J-V curves of champion devices for DC-PA and DCPA-IAHA [67].

In addition to the above methods, using covalent self-assembled monolayers and noncovalent wetting layers as HTLs in devices, namely self-assembled bilayers (SABs), is also an effective way to improve wettability [65]. The strengthening mechanism of depositing 4NH_3_ICz on Br-2PACz lies in the fact that the originally non-polar surface energy becomes polar after the addition of 4NH_3_ICz, which means that the quality of the perovskite film deposited on this interface will be higher. Although Spiro-OMeTAD is currently the most effective HTL material among p-i-n devices, insufficient doping and non-uniformity remain obstacles to improving efficiency. Therefore, some researchers have proposed adding conjugated photonic acid (CPA) to Spiro-OMeTAD to improve device efficiency and intrinsic stability. In this study, the optimization effects of BCB (monobasic acid) and BCZ (dicarboxylic acid) on Spiro-OMeTAD were compared, respectively. The increased CPA content caused a significant increase of UV-vis absorption for Spiro OMeTAD at 525 nm (Figure 9d), indicating that CPAs can better promote the oxidation of Spiro OMeTAD. It is worth noting that a (BCZ-Ag) polymer film can be formed between the phosphate group in BCZ and Ag. This can be seen from the 3d orbital XPS spectrum of Ag (Figure 8e), where the peak position of the BCZ/Ag film shifts towards the lower binding energy relative to Ag. On this basis, it is necessary to measure the breakdown voltage in order to avoid the serious impact of Ag electrode diffusion to HTL on the perovskite. In Figure 8f, it can be seen that the control group and BCB device experienced a short circuit at 3.9 V and 4.1 V, respectively, while the BCZ device only experienced a short circuit at 4.4 V due to the previously mentioned Ag BCZ film effect. The results indicate that the dicarboxylic acid structure of BCZ can significantly enhance the stability and ion fixation ability of HTL, thereby increasing the efficiency and stability of the device. The PCE of the device with BCZ added increased from 20.9% (the control) to 24.51% [66].

Another important issue with the use of SAM materials is the tendency to form micelles in solvents, which requires additional energy to decompose the micelles during film formation and lead to the generation of defects. To reduce the influence of micelles on film formation, N, N-dimethylformamide (DMF) was added into the SAM precursor solution and the strong solvent-solute interaction between DMF and carbazole groups was utilized to decompose the SAM micelles [52]. The device without co-solution treatment underwent PL quenching at around 25 s, while the device that had co-solution treatment did not undergo quenching until around 41 s under the combined action of DMF and CbzNaph (Figure 9a). During annealing, the PL strength of co-solution-treated devices begins to decrease from the first few seconds and gradually stabilizes under the action of DMF, while non-co-solution-treated devices increase the PL strength with increasing heating time due to the absence of SAM molecules forming micelles during heat (Figure 9b). The diffraction peak of the Co-SAM-based perovskite film on the (110) crystal plane is significantly higher than that of the control film (Figure 9c). The improvement of film quality reduces the density of interface traps and leads to less charge recombination. Therefore, the energy barrier during the growth process of SAM after co-solvent treatment decreases with the decomposition of micelles, and the degree of damage during annealing is also reduced, which can form a denser film. The energy alignment effect at the interface between the perovskite film and SAM is better, and the charge recombination is further suppressed.

On the basis of MeO-2PACz, Deng and his colleagues developed a novel self-assembled molecule DC-PA, which was combined with 6-(iodo-λ^5^-azanyl) hexanoic acid (IAHA) to form co-SAMs for inverted PSCs [67]. Firstly, the change in methoxy group position not only reduces the HOMO energy level of DC-PA (−4.98~−5.08 eV) but also increases the dipole from 0.12 D to 1.64 D (Figure 9d), enabling more effective electron extraction. Secondly, the results of atomic force microscopy (AFM) indicate that the perovskite film formed on the co-SAM substrate has lower roughness (Figure 9e), which means that the surface uniformity of the crystallization is higher. As well, the TRPL in Figure 9f (DCPA-IAHA) also indirectly proves that ammonium SAMs can reduce interface defects. Based on these changes, PSC devices under the action of co-SAMs achieved 23.59% PCE and improved device stability.

### 3.2. Application in Organic Solar Cells

**Figure 10:**
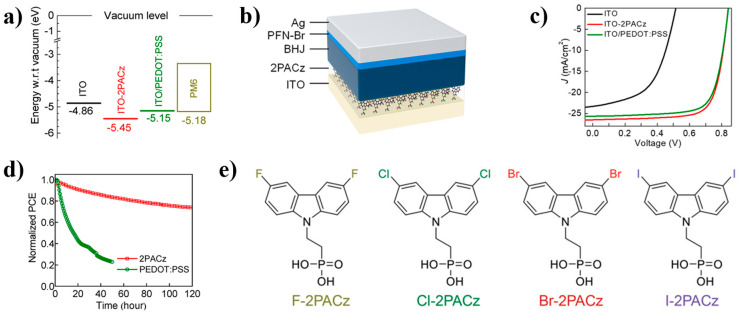
(**a**) Work function of ITO, ITO-2PACz, ITO/PEDOT:PSS, and HOMO level of PM6; (**b**) Schematic of the standard cell architectures employed; (**c**) J-V curves of PM6:N3 solar cells based on ITO, ITO-2PACz, and ITO/PEDOT:PSS [68]; (**d**) Normalized stability changes of ITO/PEDOT: PSS and ITO/2PACz under continuous illumination; (**e**) structure of F-2PACz, Cl-2PACz, Br-2PAz, and I-2PACz [27].

In addition to being widely used in PSCs, SAMs are also used in organic solar cells (OSCs). As is well known, 2PACz can change the WF of ITO (Figure 10a), generating effective hole-selective contact. By incorporating 2PACz and PEDOT:PSS into the OSCs (Figure 10b), the device performance has been significantly improved compared to bare ITO. The device efficiency has increased from 6.45% (bare ITO) to 15.94% (PEDOT:PSS) and 16.6% (2PACz). This can be seen from the J-V curve in Figure 10c. It is worth noting that although PEDOT:PSS performs as well as 2PACz in efficiency testing, its performance in stability testing is far inferior to 2PACz. Under continuous illumination of a white light-emitting diode (LED) with a light intensity of 200 mW/cm^2^, ITO/2PACz can still maintain an initial efficiency of 74% after 120 h, while ITO/PEDOT:PSS only loses 80% of its initial efficiency after 50 h (Figure 10d) [68]. On the basis of 2PACz, halogen atom substitution is also very effective in changing the work function. Lin et al. used four conventional halogen atoms (F, Cl, Br, and I) to replace the hydrogen atoms on 2PACz (Figure 10e). When the substituted molecules acted on ITO, their work functions increased from 4.73 eV to 5.68, 5.77, 5.82, and 5.73 eV, respectively [27].

**Figure 11:**
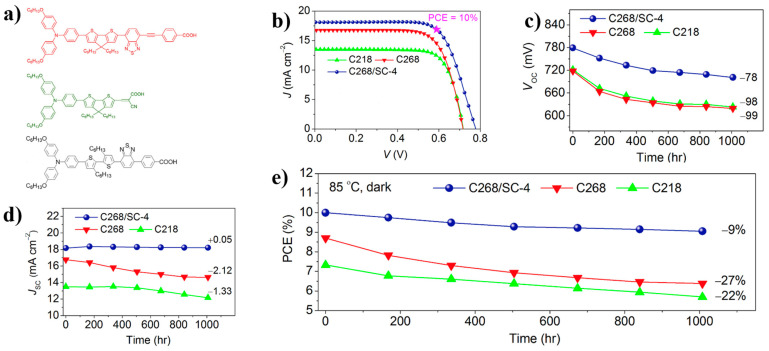
(**a**) The structure of C268 (Red), C218 (Green), and SC-4 (Black); (**b**) J-V curves under the standard AM1.5G, 100 mW cm^−2^ conditions.; (**c**,**d**) Changes in Voc and Jsc under continuous illumination; (**e**) PCE changes in dark environments [72].

In addition to novel self-assembled pyridine-like molecules such as 2PACz used in OSCs, fullerene and its derivatives also exhibit excellent performance. In early research by Stubhan, the layered structure of fullerene derivatives had a good inhibitory effect on the recombination at the interface of the electric extraction layer (EEL)/active layer. It also proves that the length of the alkane chain has almost no effect on the charge transfer process [69]. On this basis, Li et al. achieved the transition between fullerene and aromatic hydrocarbons π hybridization (Figure 11a), enhancinh electron transfer beneath fullerene derivatives and metal oxides. This newly synthesized molecule can not only alter the WF of ZnO, but also enhance the electronic extraction from BHJ to ETL and the phase transition at the top of BHJ [70].

Besides the self-assembly of macromolecules mentioned above, there are also some carboxylic acid self-assembled molecules with smaller molecular weights. Yip’s group prepared six different types of self-assembled molecules equipped with three different electrodes, Ag, Al, and Au. Through this series of molecular prepared device results, it can be seen that the interface dipole effect of BA-OCH_3_ is relatively high, so the performance of the device with interfacial modification is greatly improved [71].

### 3.3. Application in Dye-Sensitized Solar Cell

The effective SAMs are also welcome in dye-sensitized solar cells. Wang et al. reported dyes C268 with SC-4 co-adsorbed on the TiO_2_ surface, forming dense self-assembled monolayer films. C218 was employed as a comparison to explore the differences between C218 and C268, and the effects of co-adsorption between C268 and SC-4 were also studied. The structure of these three types of molecule and the J-V curves under the standard AM1.5G, 100 mW cm^−2^ conditions are shown in Figure 11a,b.

Under continuous sunlight exposure for 1000 h, the V_oc_ of the three devices decreased by 78, 98, and 99 mV (Figure 11c), respectively. In the part of J_sc_, the C268 and C218 devices decreased by 2.12 and 1.33, respectively, while the C268/SC-4 devices remained very stable (Figure 11d). In addition, stability testing in a dark environment at 85 °C, as shown in Figure 11e, showed that after 1000 h, the PCE of C268 and C218 devices decreased by 26% and 22%, respectively, while the PCE of C268/SC-4 devices only decreased by 9% [72].

## 4. Challenges and Opportunities of SAMs

### 4.1. Challenges of SAMs

SAM materials face great difficulties in observation because of their small molecule and chemical adsorption properties. Conventional characterization methods such as SEM and transmission electron microscopy are unable to observe their microstructure properly. Currently, the most common characterization methods are XPS, atomic force microscopy, and water contact angle measurements to characterize the adsorption process and atomic arrangement [23,73]. However, the specific arrangement of SAM one-way cannot be determined, which makes it impossible to observe the continuity and defect situation of SAM. If further research is needed on the effectiveness and mechanism of SAM, more surface analysis techniques are needed.

In addition, an important issue that SAM materials must consider in commercial applications is the optical and thermal stability, especially of the HTL layer located on the incident side of the light in reverse devices. In previous studies, scholars have also studied the influence of different anchoring groups on device stability. Anchoring groups with strong acidity can exacerbate corrosion of the substrate, leading to a decrease in device stability [25]. To address the issue of light stability, a molecular structure can be used to replace the alkyl links with a condensed phenylene group [74]. This structure makes it possible to separate the effective ionization regions and spaces of HOMO and LUMO, thereby improving the stability of SAM. In terms of thermal stability, adding benzene to carbazole carboxylic acids can increase the thermal decomposition temperature from 180 °C to 354 °C [53]. Therefore, in subsequent research, emphasis can be placed on these aspects to further improve the stability of the device.

### 4.2. Opportunities of SAMs

In addition to being used in PSCs, SAM materials also play an important role in some optical devices due to their excellent performance. As one of the most important optoelectronic devices at present, perovskite materials are potential materials for photodetectors due to their excellent performance. Similar to SCs, the interface of photodetectors can also be modified with SAM materials. Therefore, surface modification and passivation using SAM can improve the photo response of perovskite polycrystalline films and utilize energy level alignment to promote the separation of photo-generated excitons, thereby enhancing photocurrent [75]. Moreover, SAM materials also have great potential in light-emitting diodes (LEDs). SAM can utilize its strong interface dipole moment induction ability to form favorable band structures, promote hole input in LED devices, reduce exciton quenching, and improve luminescence performance [76].

## 5. Conclusions

This topical review started with the structure and energy levels of SAM materials, introducing the molecular characteristics of self-assembled monolayers and their applications in PSCs, OSCs, and DSSCs. In addition, the future challenges and potential research directions of SAM in the application process were also discussed. Most SAMs act as HTL in PSCs and bring significant changes, but they can also be applied in other optoelectronic devices such as photodetectors and LEDs. Although SAM materials have achieved good results in solar cells, there is still significant room for improvement in stability, especially in characterization methods, which will be the focus of future research on SAM materials. In addition, based on the research results of other scholars, we also make prospects for the application of SAM materials in other optoelectronic devices.

## Figures and Tables

**Table 1:** Partial SAM structures, types, and advantages applied to PSCs.

Structure	SAM Type	Passivated Layer (Type)	Benefits	Refs.
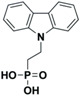 **2PACz**	Carbazole-based SAM	ITO (p)	The WF of ITO is increased, producing an efficient hole-selective contact, performing even better than PTAA.	[21]
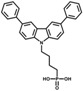 **Ph-4PACz**	Carbazole-based SAM	ITO (p)	Improves wettability and film uniformity, reduces buried interface voltage loss.	[28]
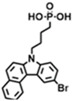 **BCBBr-C4PA**	Carbazole-based SAM	ITO (p)	Enhances interface charge transfer and suppresses nonradiative recombination losses.	[29]
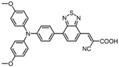 **MPA-BT-CA**	Aromatic amine-based SAM	ITO (p)	Effectively adjusts molecular orbital energy levels, modifies ITO interfaces, and effectively passivates defects in perovskite layers.	[30]
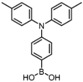 **MTPA-BA**	Aromatic amine-based SAM	ITO (p)	Reduces corrosion on ITO and significantly improves stability.	[25]
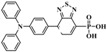 **PPA**	Aromatic amine-based SAM	ITO (n)	Passivates perovskite buried layer to improve stability.	[31]
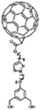 **C9**	Fullerene-based SAM	SnO_2_ (n)	Can passivate oxygen vacancy defects.	[32]
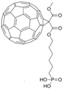 **C60-SAM**	Fullerene-based SAM	ITO (n)	Accelerates electronic extraction.	[33]
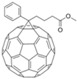 **PCBM**	Fullerene-based SAM	ITO (n)	Reduces hysteresis, improves device performance and stability.	[33]
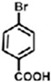 **Br-BA**	Insulating SAM	NiO (n)	Passivates surface defects on nanoparticles and enhances perovskite crystallization.	[34]
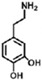 **DA-SAM**	Insulating SAM	SnO_2_ (n)	The modified interface has better carrier transport and interface electrical recombination.	[35]
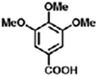 **TMBA**	Insulating SAM	ZnO (n)	Has increased built-in voltage and improved charge transfer.	[36]
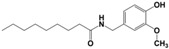 **Capsaicin**	Lewis base	Under-coordinated Pb atoms	Promotes charge transfer, suppresses defect assisted recombination and interface carrier recombination.	[37]
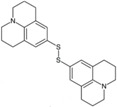 **Juls**	Disulfide	Metal electrodes	Reduces the WF of precious metal electrodes, improves the wettability of layer metal surfaces, and enhances device stability.	[38]
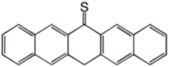 **Thioketone**	Sulfonic acid	Metal electrodes	Improves electronic coupling between contacts and semiconductors, reduces contact resistance.	[39]
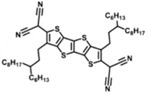 **CMUT**	Monolayer molecular crystals	Polymeric substrate	Has high mobility and can achieve low thermal activation energy thermal activation transport at 80–200K.	[40]
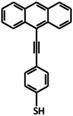 **MPEA**	Alkanethiolate	Metal electrodes	Adjusts conductivity by changing electron distribution.	[41]
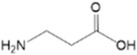 **C3**	Amino carboxylic acid	ZnO (n)	Reduces ZnO work function and increases current.	[42]
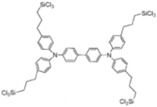 **TPD-Si_4_**	Chlorosilane-tethered	ITO (n)	Improves quantum efficiency and stability.	[43]
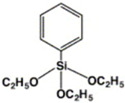 **PTES**	Silane derivatives	ITO (n)	Enhances carrier transport and improves thermal stability.	[44]
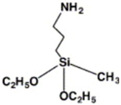 **APMDS**	Silane derivatives	ITO (n)	Enhances carrier transport and improves thermal stability with a better effect than PTES.	[44]
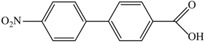 **NBCA**	Benzoic acid derivatives	ITO (n)	Enhances the work function of ITO.	[45]

## Data Availability

Data are contained within the article.
